# Periodic Fever: A Review on Clinical, Management and Guideline for Iranian Patients - Part I

**Published:** 2013-11-16

**Authors:** Zahra Ahmadinejad, Sedigeh Mansori, Vahid Ziaee, Neda Alijani, Yahya Aghighi, Nima Parvaneh, Mohammad-Hassan Mordinejad

**Affiliations:** 1 Department of Infectious Diseases, Imam Khomeini Hospital; 2 Pediatric Rheumatology Research Group, Rheumatology Research Center; 3 Department of Pediatrics, Tehran University of Medical Sciences; 4 Children’s Medical Center, Pediatrics Center of Excellence; 5 Pediatric Infectious Diseases Research Center; 6 Growth & Development Research Center, Tehran University of Medical Sciences, Tehran, Iran

**Keywords:** Periodic Fever, Familial Mediterranean Fever, PFAPA, Hyper IgD, TRAPS

## Abstract

Periodic fever syndromes are a group of diseases characterized by episodes of fever with healthy intervals between febrile episodes. The first manifestation of these disorders are present in childhood and adolescence, but infrequently it may be presented in young and middle ages. Genetic base has been known for all types of periodic fever syndromes except periodic fever, aphthous stomatitis, pharyngitis, and cervical adenitis (PFAPA). Common periodic fever disorders are Familial Mediterranean fever (FMF) and PFAPA. In each patient with periodic fever, acquired infection with chronic and periodic nature should be ruled out. It depends on epidemiology of infectious diseases. Some of them such as Familial Mediterranean fever and PFAPA are common in Iran. In Iran and other Middle East countries, brucellosis, malaria and infectious mononucleosis should be considered in differential diagnosis of periodic fever disorders especially with fever and arthritis manifestation. In children, urinary tract infection may be presented as periodic disorder, urine analysis and culture is necessary in each child with periodic symptoms. Some malignancies such as leukemia and tumoral lesions should be excluded in patients with periodic syndrome and weight loss in any age. After excluding infection, malignancy and cyclic neutropenia, FMF and PFAPA are the most common periodic fever disorders. Similar to other countries, Hyper IgD, Chronic Infantile Neurologic Cutaneous and Articular, TRAPS and other auto-inflammatory syndromes are rare causes of periodic fever in Iranian system registry. In part 1 of this paper we reviewed the prevalence of FMF and PFAPA in Iran. In part 2, some uncommon auto-inflammatory disorders such as TRAPS, Hyper IgD sydrome and cryopyrin associated periodic syndromes will be reviewed.

## Introduction

Periodic fever syndromes are a group of diseases characterized by episodes of fever with healthy intervals between febrile episodes. These disorders are self-limited and improve spontaneously without specific therapy. Episodes of fever are usually associated with presence of inflammation in different parts of the body such as peritoneum, pleural spaces, testis, etc. with elevated serum acute phase reactants level. These diseases should be differentiated from infections, malignancies and other autoimmune disorders^[^^1^^]^. Existence of family history of periodic fever and prolonged illnesses are diagnostic clues of these disorders.

 At least eight hereditary periodic fever syndromes have been known so far which are common in fever with cutaneous manifestations, and musculoskeletal involvement^[^^2^^]^, each of them having specific characteristics. Fever and symptoms occur due to the increment of inflammation which is caused by mutation in genes. Most of these disorders result from gene mutations that cause defect in production of some proteins which have important role in control of inflammation and apoptosis.

 Usually there are symptoms of the disease for a long time prior to the definite diagnosis. Mostly fever attacks start during childhood, but sometimes they may start in adolescence or in adulthood. Clinical examinations are helpful for diagnosis which is confirmed by specific genetic testing^[^^3^^]^. 

 After a brief review of pathogenesis, we present a new approach to the periodic fever in Iranian patients based on epidemiology of infectious and auto-inflammatory disorders in Iran. In addition, we will explain the most important clinical and laboratory data of different types of periodic fever syndromes based on our experience.

***Definition and Clinical Manifestations***

Periodic fever disorders are a group of recurrent and episodic disorders with fever as a main complaint. Arthritis or arthralgia, abdominal or chest pain due to serositis, skin rash, and oral ulcers are other common presentations of periodic fever syndromes, although these symptoms can be found in infectious diseases with prolonged or recurrent fever^[^^4^^,^^5^^]^. Headache, seizure, aseptic meningitis, development delay as well as adenopathy, organomegaly and cardiac involvement are uncommon findings in these disorders. Renal involvement is uncommon but in some types, renal failure due to amyloidosis may occur. Periodic fever is named when there are 3 episodes of fever in at least 3-6 months with 7 days asymptomatic period between each episode^[^^5^^]^. Rarely, periodic fever syndrome without fever has been reported^[^^6^^,^^7^^]^. In this situation, one of the other symptoms is repeated periodically. For this reason, we believe that periodic fever should be considered as a diagnosis in a patient, who shows similar problem repeatedly. Some periodic fever syndromes occur in regular intervals especially periodic fever, aphthous stomatitis, pharyngitis, cervical adenitis (PFAPA) and cyclic neutropenia, however, Familial Mediterranean Fever (FMF) and hyperimmunoglobulin D (hyper IgD) generally have regular patterns. On the other hand, auto-inflammatory syndromes in infancy such as chronic infantile neurologic cutaneous and articular (CINCA) syndrome, Muckle-Wells syndrome, familial cold urticaria and tumor necrosis factor receptor–associated periodic syndrome (TRAPS) usually have irregular patterns^[^^8^^,^^9^^]^.

***Pathogenesis***

Periodic fever syndrome disorders are categorized under the term monogenic auto-inflammatory syndromes. The characteristic of these disorders is episodic attacks of systemic inflammations without presence of infection or auto-antibodies^[^^2^^,^^10^^,^^11^^]^. The pathogenesis of these disorders is disregulation of inflammation control due to mutations of genes coding for some proteins with regulation role^[^^11^^]^. It has been believed that dysregulation of innate immune response and abnormalities in activity of interleukin1 and pro-inflammatory cytokines may contribute to fever production and systemic inflammation^[^^2^^,^^12^^]^. Innate immune cells such as macrophages, neutrophils, and monocytes are involved^[^^12^^]^. Unlike adaptive immunity, innate immunity is programmed genetically^[^^13^^]^ and most of these diseases are caused by mutation in genes which make proteins participant in inflammatory response^[^^14^^]^. 

 It is thought that mutate proteins lead to increased or prolonged secretion of pro-inflammatory cytokines^[^^15^^]^. Activation of caspase-1 and the release of IL-1β are the end point of pathophysiologic cycle of these disorders^[^^16^^]^. Treatment with anti-IL1 drugs is mandatory^[^^15^^]^. 

***Approach to Periodic Fever***

In a patient with periodic fever, acquired infection with chronic and periodic nature should be ruled out. It depends on epidemiology of infectious diseases. In Iran and other Middle East countries, brucellosis, Malaria, infectious mononucleosis and borelliosis should be considered in differential diagnosis of periodic fever disorders especially with fever and arthritic manifestation^[^^17^^-^^19^^]^. Malaria and borrelliosis can be ruled out by evaluation of peripheral blood smear and brucellosis and Lyme disease can be excluded by serological studies or blood culture. In children, urinary tract infection may present as a periodic disorder, so urine analysis and culture are mandatory in a child with periodic symptoms^[^^4^^]^. Some malignancies such as leukemia and tumoral lesions should be excluded in patients with periodic syndromes and weight loss in any age and weight gain disorder or malaise in children^[^^9^^,^^20^^]^. For this reason, abdominal ultrasound and chest X-ray are necessary and bone marrow aspiration in selected patients is recommended. Table 1 shows paraclinical evaluation in suspected patients with periodic fever syndromes. 

 After ruling out infection and malignancies, auto-inflammatory disorders should be considered. However, physician needs to reevaluate each patient with periodic disorder for non-inflammatory syndromes. 

 In Iran after excluding infections, malignancy and cyclic neutropenia, FMF and PFAPA are the most common periodic fever disorders. Similar to other countries, hyper IgD, CINCA syndrome, TRAPS and other auto-inflammatory syndromes are rare causes of periodic fever disorders in our system registry of periodic fever in Iran. Fig. 1 and Fig. 2 show clinical approach to periodic fever in Iranian patients in Periodic Fever Clinic in Children’s Medical Center, based on the epidemiology of periodic fever in this country.

***Familial Mediterranean Fever (FMF)***

FMF or recurrent hereditary polyserositis is the most common disease among hereditary periodic fever syndromes, and it is an autosomal recessive disorder^[^^21^^]^. 

**Algorithm 1:**
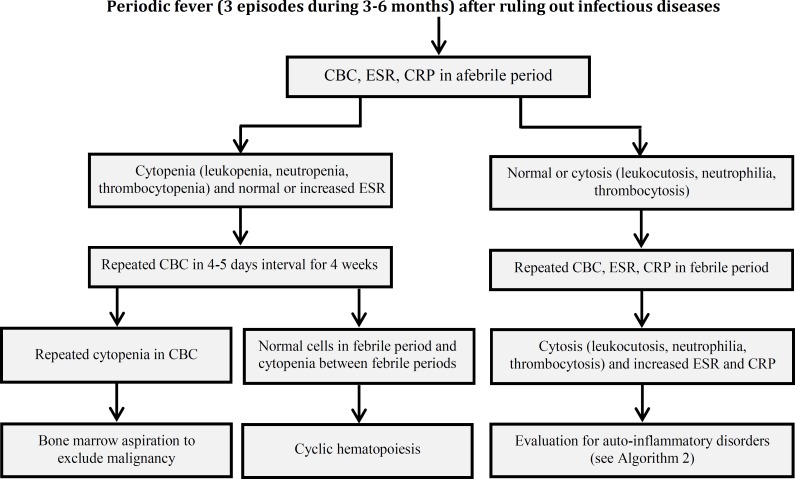
First step to approach to Periodic Fever

CBC: Complete Blood Cells; ESR: Erythrocyte Sedimentation Rate; CRP: C-Reactive Protein

**Algorithm 2:**
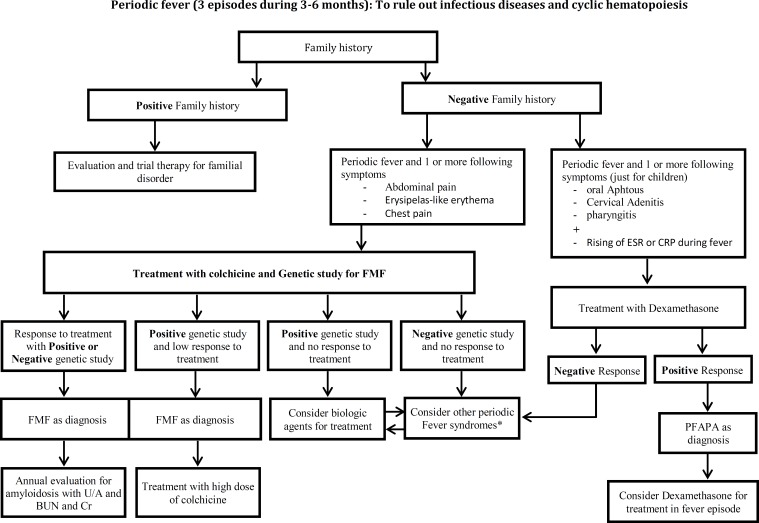
Second step to approach to Periodic Fever

* It can be as a coincidence of FMF and other periodic fever disorders (such as PFAPA)

FMF: Familial Mediterranean Fever ; PFAPA: Periodic Fever, Aphthus stomatitis, Pharyngitis, Cervical Adenitis; U/A: Urinaly sis;

BUN; Blood Urea Nitrogen; Cr: Creatinine; ESR: Erythrocyte Sedimentation Rate; CRP: C - Reactive Protein

**Algorithm 2 (continue):**
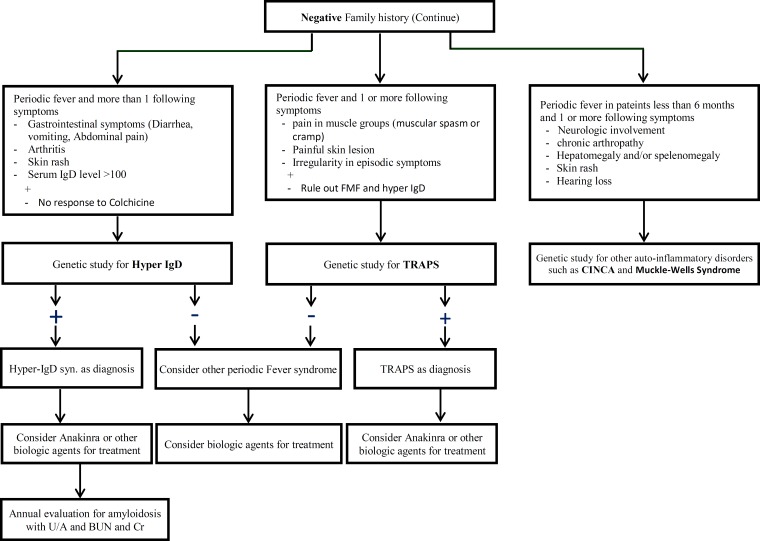
Second step to approach to Periodic Fever

IgD: Hyperimmunoglobulin D; TRAPS: Tumor Necrosis Factor Receptor–Associated Periodic Syndrome;

CINCA: Chronic Infantile Neurologic Cutaneous a nd Articular Syndrome

**Table 1:** Laboratory tests for patients with periodic fever

**Study **	**Indication**	**Time**
**CBC, ESR, CRP**	All patients	At least 2 times in febrile and afebrile period
**Chest X-ray**	All patients	In febrile period
**Abdominal ultrasound**	All patients	In febrile period
**Serum Igs**	All patients	No difference
**Wright, Coombs-Wright**	In endemic region	At least once in febrile period
**EBV serology**	All patients with sore throat and lymphadenopathy	In febrile period
**Peripheral blood smear**	In endemic region	At least once in febrile period
**Blood culture**	All patients	At least once in febrile period
**Throat culture**	All patients with sore throat	In febrile period
**Urine analysis and culture**	All children	At least once in febrile period
**Stool exam**	All children	At least once, no difference
**Bone marrow aspiration**	In selected patients with cytopenia, bone pain, weight loss, abdominal and/or mediastinal adenopathy	No difference
**Liver function tests**	All patients	At least once in febrile period
**FANA**	All children	At least once, no difference

*CBC: Complete Blood Cells; ESR: Erythrocyte Sedimentation Rate; CRP: C-Reactive Protein; Ig: Immunoglobulin;

EBV: Epstein Barr Virus; FANA: Fluorescent Antinuclear Antibody Test

***Epidemiology: ***This disease is seen most frequently in people from Mediterranean area such as Jews, Arabs, Turks and Armenians, but it is sometimes reported from all over the world. Prevalence of FMF is 1:200 to 1:1000^[^^8^^,^^22^^]^. Although FMF is a autosomal receive disorders, positive family history is present in less than 50% of patients^[^^9^^,^^23^^,^^24^^]^. This disease is slightly more common in women perhaps due to the influence of sex hormones. Sometimes fever attacks disappear in pregnancy and return after delivery. Rarely infertility may occur due to defect in ovulation and adhesion in pelvic peritoneum. Some complications of FMF such as renal amyloidosis are more common in males^[^^21^^]^.

***Pathophysiology: ***The disease is characterized by periodic fever that occasionally occurs at regular intervals, usually with neutrophil induced serositis. The responsible gene for the disease is MEFV that is located on short arm of chromosome 16^[^^2^^]^. This gene produces a protein called Pyrin or Marenostrin that is found in neutrophils and plays an important role in reduction of inflammation^[^^25^^]^. Mutation in this gene leads to defection in production of these proteins and onset of inflammatory cycle. In addition to gene mutation, environmental factors are important and in many patients there is a trigger factor such as emotional stress, infection, extreme physical exercise, fatigue, trauma and menses^[^^12^^,^^21^^,^^26^^,^^27^^]^.

***Clinical Manifestations: ***Familial Mediterranean fever usually begins in early childhood and 60-90% of patients are younger than 20 years^[^^12^^,^^21^^]^. The mean age of patients at onset of symptoms is 2.5 years. Mean age of patients at the time of diagnosis is 4 years. Very rarely it may occur after 30 years of age^[^^28^^]^. 

 Classical symptoms of FMF are periods of fever and abdominal pain. Frequency of these periods is variable from once in a week to several months. Fever is the most common symptom and it is seen in almost all cases^[^^8^^,^^12^^,^^23^^]^. The fever is usually short-term and usually takes 1 to 3 days and it resolves without treatment^[^^8^^,^^12^^]^. Recurrent oral aphthae may occur without correlation with fever attacks. Fever may initially be the only symptom for many years without another sign in children^[^^8^^,^^12^^,^^25^^]^. 

 Abdominal pain is the second most common symptom that is seen in more than 80% of patients^[^^9^^,^^23^^,^^29^^]^. It is usually generalized and has an acute onset, is usually associated with vomiting and diarrhea and sometimes with guarding and rebound tenderness that mimicks acute appendicitis but prophylactic appendectomy for prevention of misdiagnosis is not recommended^[^^8^^,^^21^^,^^25^^,^^30^^]^. Sometimes this pain leads to laparotomy before the definite diagnosis^[^^30^^]^. Rarely a condition called chronic abdominal disease results from recurrent inflammation leading to peritoneal adhesion and subclinical inflammation between fever attacks^[^^21^^]^. 

 Serositis in different cavities like abdomen (peritonitis), chest (pleuritis and pericarditis), joints (arthritis and sinovitis) is another clinical presentation of FMF^[^^9^^,^^12^^,^^31^^]^. Another common symptom is febrile chest attack that occurs in about 30% and it characterized with painful breathing^[^^8^^,^^21^^,^^23^^,^^29^^]^. It is usually unilateral and increases with inspiration. On physical examination decrease of breath sounds may be present and in chest radiography minimal pleural effusion or pleural thickening may be observed^[^^25^^]^. Patients with homozygous mutation of M694V experience more episodes of pleural attacks^[^^21^^]^.

 Arthritis is seen in 70% of patients and is usually monoarthritis, nondestructive and occurs in large joints of lower extremities such as ankle, knee and hip^[^^25^^,^^32^^]^. The involved joint is red, tender and swollen resembling septic arthritis. Synovial fluid is inflammatory in which neutrophil count is elevated but microbial culture is negative^[^^32^^]^. Arthritis disappears with nonsteroidal anti-inflammatory drugs (NSAIDs). The period of this arthritis is longer than other manifestations of FMF and rarely takes more than one month. Arthritis in adults may be destructive and lead to joint replacement^[^^25^^]^.

 Muscular pain is observed in 10% of patients with FMF, and is one of the debilitating manifestations that sometimes last several weeks in patients treated with NSAIDs, and may be associated with abdominal pain without peritonitis^[^^21^^]^. Acute phase reactants and sedimentation rate are higher in myalgia than in other manifestations of FMF. Muscular enzymes and muscle biopsy are normal^[^^2^^]^. Myalgia responds well to treatment with corticosteroids, but not to colchicine. It must be differentiated from colchicine-induced myopathy^[^^8^^,^^21^^]^. 

 Erysipelas like erythema (ELE) is found in less than 30% of patients^[^^23^^,^^27^^,^^29^^,^^33^^,^^34^^]^. This rash is sometimes associated with arthritis and is usually observed in lower extremities, the skin of which is red, warm and tender, sometimes being difficult to differentiate from cellulitis. Pathologic feature of these lesions is inflammation in superficial dermis and perivascular infiltration without vasculitis. Direct immunofluorescence shows C3 deposits in the wall of small vessels^[^^8^^]^. Incidence of Henoch-Schoenlein purpura (HSP) and polyarteritis nodosa (PAN) are more prevalent in patients with FMF and the age of onset is younger than in other people^[^^8^^,^^14^^]^. 

 Renal amyloidosis is the worst complication of FMF that is seen in untreated patients. Persistent inflammation causes amyloid deposition on kidneys and can lead to nephrotic syndrome and renal impairment^[^^14^^]^. This condition progresses in several years. We discuss about risk factors of amyloidosis later in prognosis section. Scarcely, renal insufficiency may be the initial presentation of the FMF (phenotype II)^[^^35^^]^. Sometimes transient microscopic hematuria is one of the findings^[^^8^^]^. Treatment with colchicine may prevent progression to amyloidosis^[^^36^^,^^37^^]^.

 Other less common manifestations of FMF are acute scrotum (involvement of tunica vaginalis), acute pericarditis, thyroiditis, meningitis and splenomegaly^[^^8^^,^^9^^,^^27^^,^^38^^,^^39^^]^. 

***Laboratory findings:*** There are no specific laboratory findings during and between febrile attacks in FMF. Leukocytosis, ESR, C-reactive protein and fibrinogen are usually elevated during febrile episodes^[^^4^^,^^21^^,^^23^^,^^31^^]^. Serum amyloid A (SAA) is an acute-phase reactant and a good marker for diagnosis of FMF between febrile attacks or in patients without febrile attacks, although the specificity of SAA is low^[^^40^^]^. In addition, SAA can be used in amyloidosis suspicion. The risk for amyloidosis increases when SAA is elevated^[^^40^^]^. It also can be used for adjustment of colchicine dose^[^^40^^]^. There is a high correlation between SAA and CRP level. So when SAA is not available, CRP can be used for amyloidosis between febrile attacks^[^^40^^]^. 

***Diagnosis and differential diagnosis:*** Diagnosis of FMF is based on clinical manifestation and exclusion of other diseases, but definite diagnosis needs genetically confirmation and finding of mutation in MEFV gene. Irritable bowel syndrome, recurrent infections, functional abdominal pain, and other periodic fever including: hyper-IgD immunoglobulinemia (HIDS), familial Hibernian fever and Marshall's syndrome should be excluded before diagnosis of FMF^[^^8^^,^^26^^]^. 

 For many years diagnosis of FMF was based on clinical criteria and exclusion of other differential diseases. However as we mentioned previously, nowadays confirmation of FMF is based on genetic study and mutation in MEFV gene.

 There are some clinical criteria to suggest the diagnosis of FMF. Tel Hashomor criteria are most often applied in adults^[^^41^^]^ but its specificity in children is low^[^^23^^,^^29^^]^. The criteria are classified into two groups of major and minor criteria. Major criteria include: (1) recurrent fever plus serositis, (2) secondary amyloid A amyloidosis, and (3) response to treatment with colchicine. Minor criteria include: (1) recurrent fever, (2) erysipelas-like erythema (ELE) and (3) familial history of FMF. The presence of two major criteria or one major criterion plus two minor ones leads to definite diagnosis^[^^29^^,^^41^^]^. On the other hand, presence of a major criterion plus a minor criterion suggests probable diagnosis^[^^21^^]^.

 The clinical criteria which are introduced by Yalcinkaya et al are as follows: fever (axillary temperature >38^o^C , 6-72 hours of duration, ≥3 attacks) abdominal pain (6-72 hours of duration, ≥3 attacks), Chest pain (6-72 hours of duration, ≥3 attacks), arthritis (6-72 hours of duration, ≥3 attacks, oligoarthritis), and family history of FMF^[^^23^^]^.

 Genetic study provides the facility to confirm FMF in difficult cases with atypical signs, late onset disease and negative family history of FMF and wider indications for molecular approach may result in more frequent diagnosis of FMF^[^^42^^]^. Nowadays, more than 80 mutations have been reported in FMF patients, the majority being located in exon 10^[^^26^^,^^43^^,^^44^^]^. Five common mutations are M694V, M680I, V726A, M694I, and E148Q that account for 85% of patient suffering from FMF^[^^4^^,^^24^^,^^26^^,^^33^^,^^34^^]^. Onset of the disease in patients with homozygous mutation occurs at earlier age^[^^29^^]^. M694V is the most popular mutation and patients with this mutation are more likely to suffer from the severe form of FMF and higher prevalence of arthritis and progress to amyloidosis^[^^44^^]^. 

***Treatment: ***The drug of choice for FMF is colchicine that is also used for prevention. It is recommended to use this drug for a long time. A minority group of patients do not respond to colchicine may be due to non-adherence^[^^21^^,^^27^^]^. Starting dose is 1mg/day and it may be increased to 1.5 to 2 mg/day until remission is achieved^[^^8^^]^. Based on weight or body surface area, the colchicine dose is 0.03±0.02 mg/kg/day and 1.16±0.45 mg/m^2^/day, respectively^[^^8^^]^. In children younger than 5 years, higher doses of colchicine, 0.07 mg/kg/day or 1.9 mg/m^2^/day, may be required^[^^8^^]^. The most common side effects of colchicine are diarrhea and nausea which are seen rarely. Fetal malformation from colchicine has not been reported^[^^8^^]^. 

 In colchicine resistance cases interferon-alpha may be helpful^[^^26^^,^^28^^]^. In patients with incomplete remission with colchicine, or in whom amyloid A level is high despite treatment with colchicine, IL1 blockade (Anakinra) may be effective^[^^12^^,^^16^^,^^31^^]^. Other drugs such as NSAIDs and corticosteroids have limited indication for treatment of FMF. Myalgia responds to treatment with corticosteroids, but not colchicines. NSAIDs are used for treatment of arthralgia^[^^21^^]^. Febrile attacks do not respond to and are not preventable by NSAIDs or steroids.

**Severity Scoring:** The severity of FMF can be predicted by Pras’s severity-scale scores (Table 2)^[^^45^^]^. This scoring can be used for establishing a treatment and follow-up strategy. In this system, mild severity is defined with a score of 2-5 points, moderate severity with a score of 6-10, and severe disease has a score more than 10. New FMF severity score was suggested by Mor et al with more sensitivity and speciﬁcity (>92%) (Table 3)^[^^46^^]^. Patients with severe FMF should be treated with high dose colchicine and should be followed closely. 

***Prognosis:*** Generally, prognosis of FMF is related to subsequent amyloidosis. Before initiating colchicine therapy, the incidence of amyloidosis was near 50%^[^^12^^]^, but it has decreased to less than 30%. The effect of several factors for development of amyloidosis has been assessed in different studies. Some authors believed there is an association between severity of FMF and its prognosis^[^^46^^]^. All factors evaluated for disease severity can play a role in FMF prognosis^[^^46^^]^. Although some investigators believed that the severity of FMF does not play a major role in development of amyloidosis in FMF^[^^32^^]^. Patients 

**Table 2:** Pras’s severity-scale scores for Familial Mediterranean fever^[^^45^^]^

**Variable**		**Score**
**Age of onset **	Younger than 5 years 5-10 years 10-20 years 20 years or older	3 points 2 points 1 point 0 point
**Frequency of attacks** ** (number per month) **	More than 2 1-2 Less than 1	3 points 2 points 1 point
**Colchicine dosage to control attacks (tablets per day) **	More than 4 (no response) 4 3 2	4 points 3 points 2 points 1 point
**Arthritis **	Protracted Acute	3 points 2 points
**Erysipelas like erythema **	Present	2 points
**Amyloidosis **	Present Phenotype II	3 points 4 points

with low compliance to colchicine therapy are in risk for renal amyloidosis^ [^^8^^,^^21^^,^^36^^,^^37^^]^. There is an association between low compliance for treatment and continuous inﬂammation (number and duration of fever attacks). Genetic predisposition is one of the other important factors in development of amyloidosis^[^^36^^]^. Mutation of M694V has a correlation with amyloidosis even in patients without fever^[^^44^^,^^47^^]^.

 Other factors which can decrease the risk of amyloidosis include: earlier treatment and continuous therapy with colchicine^[^^37^^]^. It seems that environmental factors such as geography play a role in development of amyloidosis^[^^12^^]^. Amyloidosis is more common in North Africans Jews, Armenians, and Turks with M694V homozygous and positive family history of amyloidosis. This complication is less seen in Iraqi Jews, American Armenians, Arabs and Iranians^[^^7^^,^^27^^]^. 

 Sometimes serum amyloid A level during the attack-free periods is elevated, and high level of amyloid A level may be the first symptom of renal amyloidosis, so monitoring serum amyloid A level may be helpful to predict the progress of renal amyloidosis^[^^40^^]^.

 Late onset disease may have a milder clinical presentation and has a better prognosis^[^^26^^]^. Amyloidosis rarely occurs in adults with late onset FMF^[^^28^^,^^40^^]^.

**Familial Mediterranean fever in Iran: **People with different ethnicities live in Iran and consanguineous marriage is common between Iranian people. These leads to more frequent diagnosis of genetic disorders like FMF in this country. The disease is seen sporadically in different parts of Iran^[^^7^^]^, however, it seems that Iranian Azeri Turks are more prone to FMF^[^^33^^]^. Demographic and clinical presentations of patients with FMF in Iran are comparable with those of other populations (Table 4). Consanguineous marriages (<40%) and disease occurrence in siblings (33%) are common in Iranian patients^[^^33^^,^^34^^]^.

**Table 3:** Second set of criteria for Familial Mediterranean fever severity score^[^^46^^]^

**Criteria**
1. > 1 site in a single attack (In at least 25% of attacks) 2. > 2 sites in the course of the disease 3. > 2 mg/day colchicine to achieve remission 4. > 2 pleuritic attacks during the course of the disease 5. > 2 Erysipelas-like erythema attacks during the course of the disease 6. Age of onset <10 years.

Severe disease >3 criteria; intermediate disease 2 criteria; mild disease < 1 criterion

**Table 4:** Comparison of clinical characteristics of Familial Mediterranean fever in Iran with other countries

**Country [Reference No]**	**Mean age at the onset of symptoms **	**Frequency of clinical findings**				
		**In >70% of patients**	**In >20% and <70% of patients**	**In <20% of patients**	**Prevalence of amyloidosis**	**The most prevalent mutations**
**Iran/ Tehran ** ^[^^33^^]^	11.4 (1 month- 28 years)	Fever, Abdominal pain	Chest pain	Erysipelas-like erythema	5.6%	M694V, M680I
**Iran/ Tabriz ** ^[^^34^^]^	18.5 (2-66 years)	Abdominal pain, Fever	Arthritis and chest pain	Erysipelas-like erythema	4%	M694V, V726A
**Iran/ Tehran ** ^[^^7^^]^	49.2 mo (2 mo-15 yrs)	Fever, Abdominal pain	Joint pain and chest pain	Erysipelas-like erythema and bone pain	0	M694V, V726A
**Iran/ Semnan ** ^[^^28^^]^	29 ±7.8	Fever, Abdominal pain	Chest pain, Scrotal pain, Headache	Arthritis and Erysipelas-like erythema	Not reported	Not studed
**Japan** ^[^^48^^]^	--	Fever	Abdominal pain, chest pain, Arthritis	Erysipelas-like erythema	3.7%	M694I/ E148Q
**Turky** ^[^^49^^]^	Range: 1-12	Fever, Abdominal pain, Arthritis	Chest pain	Vvasculitis rash	3.5%	M694V, M680I
**Arabs/Egypt ** ^[^^50^^, ^^51^^]^	6.9±2.8	Fever, Abdominal pain,	Arthralgia, Myalgia and Operation	Arthritis, chest pain	Not reported	V726A/M694V
**Armenia** ^[^^24^^]^	10.8±8.4	Fever, Abdominal pain, Chest pain, Arthritis	--	--	21.2%	M694V, M680I

Fever and abdominal pain are the most common clinical findings occurring in more than 85% of patients while erysipelas-like erythema is reported in less than 10%^[^^7^^,^^33^^,^^34^^]^. 

 Molecular approach for diagnosis of FMF is rapidly expanding in Iran and some referral laboratories for genetic study such as the laboratory of Children’s Medical Center in Tehran. In Iranian people, M694V is the most common mutation^[^^33^^,^^34^^]^. Other common mutations in Iranians are M680I, V726A, E148Q and M694I. Recently, in a study on a group of Iranian patients in our center, we found 2 rare mutations in 2 patients in exon 10 in codons^[^^52^^]^. Most Iranian patients have good (60%-100%) response to colchicine ^[^^7^^,^^28^^,^^33^^,^^34^^]^. Amyloidosis has been reported in 0-5.6% of Iranian FMF patients^[^^7^^,^^33^^.^^34^^]^.

***Periodic fever, Aphthous stomatitis, Pharyngitis, Cervical adenitis (PFAPA)***

Marshall's syndrome or Periodic fever, aphthous stomatitis, pharyngitis, cervical adenitis which stands for PFAPA is a sporadic syndrome characterized by recurrent febrile disease with symptoms in head and neck^[^^4^^,^^8^^,^^53^^]^.

**Clinical Pearls in FMF**

- It is an autosomal recessive disorder and the most common disease among HPFS.
- The MEFV Gene which is responsible for the disease is located on short arm of chromosome 16.
- It usually begins in early childhood.
- Common clinical manifestations are: recurrent fever, abdominal pain, arthritis and erysipelas-like erythema.
- Renal amyloidosis is the worst complication of FMF.
- Definite diagnosis of FMF is based on finding of the mutation in MEFV gene.
- The main drug for treatment of FMF is colchicine.

**Table 5:** Clinical criteria of Periodic Fever, Aphthus stomatitis, Pharyngitis, Cervical Adenitis

Regulatory recurring fevers with an early age of onset (<5 years of age) Symptoms in the absence of upper respiratory tract infection with at least one of the following clinical signs: a) aphthous stomatitis b) cervical lymphadenitis c) pharyngitis Exclusion of cyclic neutropenia, completely asymptomatic interval between episodes, normal growth and development.

***Epidemiology: ***The age of onset is before 5 years^[^^4^^,^^9^^,^^31^^,^^53^^]^ and this disease is slightly more common in males^[^^4^^,^^31^^]^.

***Etiology and Pathophysiology: ***The etiology of the illness is still unknown, but it is thought that impairment in regulation of cytokine production may be involved in pathogenesis of PFAPA^[^^4^^,^^8^^,^^31^^]^. Several cytokines such as IFN, TNF, and IL-6 are elevated in febrile episodes^[^^8^^]^. It seems that an abnormal immune response to an antigen in oral cavity or tonsils is responsible for symptoms. Pathologic findings in tonsils are nothing but just nonspecific chronic inflammation^[^^54^^]^.

***Clinical Manifestations: ***PFAPA usually occurs in regular intervals and episodes of fever every 2 to 12 weeks, but when the child gets older the intervals of the disease increase^[^^9^^,^^31^^,^^53^^]^. Patients feel well between episodes of the illness. The onset of the disease is along with malaise and then an abrupt fever which may rise up to 40^o^C. Exudative tonsillitis is seen in most patients, and aphtous stomatitis is seen in 70% of patients. Cervical adenitis presents in 88-100% of patients. Chills, sweating, headache, and myalgia are common^[^^8^^,^^53^^]^. Sometimes mild hepatospleno-megaly and arthralgia may be seen^[^^8^^]^. Symptoms of the illness usually last for 3-4 days and resolve slowly^[^^4^^,^^31^^]^ without sequels^[^^9^^]^. 

***Laboratory findings:*** Laboratory findings are mild leukocytosis, elevated ESR and CRP^[^^8^^,^^9^^,^^31^^]^, and high level of serum IgD in 66% of patients and elevated IgE level in 50% of patients^[^^8^^]^. Radiologic findings are normal in all patients^[^^8^^]^. 

***Diagnosis and differential diagnosis: ***The diagnosis is made clinically with exclusion of other diseases and with significant response to single dose of corticosteroids^[^^53^^]^. In 1999 “Thomas et al” ^,^diagnostic criteria were determined in order to facilitate diagnosis of PFAPA (Table 5)^[^^53^^]^. PFAPA should be differentiated from other periodic febrile illnesses such as recurrent tonsillitis, hyperglobulinemia D syndrome, cyclic neutron-penia and infectious diseases^[^^8^^,^^12^^,^^53^^]^.

***Treatment:*** Treatment with antibiotics and NSAIDs is ineffective and PFAPA dramatically responds to a single dose of prednisolone 2mg/kg or 0.3mg/kg of bethamethasone^[^^8^^]^. They are the first line therapy but the treatment may be associated with decreased intervals of the disease^[^^9^^]^ and prednisolone cannot prevent subsequent episodes^[^^53^^]^. Fever subsides within 2 to 4 hours but other symptoms such as aphthous stomatitis disappear slower than the fever^[^^8^^]^. Cimetidine is used for prevention of episodes in some centers^[^^9^^]^. Recently it is proven that tonsillectomy with or without adenoidectomy resolves symptoms of the disease in more than 90% of cases and improves symptoms in 4.6% of cases^[^^53^^,^^54^^]^ but not all experts do recommend it. 

***Prognosis: ***Prognosis of PFAPA is very excellent. Affected children have normal development and growth^[^^8^^]^. No morbidity or mortality has been reported up to now in PFAPA patients. Unlike other periodic fevers, amyloidosis or chronic organ involvement is not a complication of PFAPA, but there is a case report on IgA nephropathy in a child after 5 years periodic fever and PFAPA. Exacerbation of hematuria has been reported in this case during fever attacks. Treatment with methyl prednisolone pulse therapy and immunosuppressives improved urine findings, but successful treatment for control of PFAPA episodes and IgA nephropathy was tonsil-ectomy^[^^55^^]^. 

**Clinical Pearls in Periodic Fever, Aphthous stomatitis, Pharyngitis, Cervical Adenitis**

- PAFAP is a periodic fever with unknown pathophysiology.
- It usually affects children younger than 5 years old.
- Prognosis is good without any prolonged complications.

***PFAPA in Iran:*** Although there is no report on characteristics of PFAPA in Iranian patients, it seems not to be rare in Iran. An unpublished report in our system registry shows PFAPA diagnosis in nearly 20% of patients with periodic fever. In a recent report from Iran, 30% of patients with PFAPA had MEFV gene mutations, although these mutations do not have any effect on presentation and course of PFAPA^[^^56^^]^.

 Other periodic fever syndromes and auto- inflammatory disorders include TRAPS, hyper IgD sydromes and cryopyrin (CINCA or NOMID, Muckle-Wells syndrome, familial cold urticaria), PAPA (pyogenic arthritis, pyoderma gangraenosum, cystic acne), CRMO (chronic recurrent multifocal osteomyelitis), DIRA (deficiency of the interleukin-1–receptor antagonist) and Majeed syndrome will be discussed in part II of this paper.
